# Score for the Overall Survival Probability of Patients With First-Diagnosed Distantly Metastatic Cervical Cancer: A Novel Nomogram-Based Risk Assessment System

**DOI:** 10.3389/fonc.2019.01106

**Published:** 2019-11-05

**Authors:** Shilong Zhang, Xin Wang, Zhanming Li, Wenrong Wang, Lishun Wang

**Affiliations:** ^1^Minhang Hospital, Fudan University, Shanghai, China; ^2^Institute of Fudan-Minhang Academic Health System, Minhang Hospital, Fudan University, Shanghai, China; ^3^Department of Acupuncture and Moxibustion, Central Hospital of Shanghai Xuhui District, Shanghai, China; ^4^Faculty of Physical Education, Shandong Normal University, Jinan, China

**Keywords:** nomogram, metastatic cervical cancer, SEER, overall survival, decision curve analysis

## Abstract

**Background:** Metastatic cervical cancer (mCEC) is the end stage of cervical cancer. This study aimed to establish and validate a nomogram to predict the overall survival (OS) of mCEC patients.

**Methods:** We investigated the Surveillance, Epidemiology, and End Results (SEER) database for mCEC patients diagnosed between 2010 and 2014. Univariate and multivariable Cox analyses was performed to select the clinically important predictors of OS when developing the nomogram. The performance of nomogram was validated with Harrell's concordance index (C-index), calibration curves, receiver operating characteristic curve (ROC), and decision curve analysis (DCA).

**Results:** One thousand two hundred and fifty-two mCEC patients were included and were divided into training (*n* = 880) and independent validation (*n* = 372) cohorts. Age, race, pathological type, histology grade, radiotherapy, and chemotherapy were independent predictors of OS and used to develop the nomogram for predicting 1- and 3-year OS. This nomogram had a C-index of 0.753 (95% confidence interval [CI]: 0.780–0.726) and 0.751 (95% CI: 0.794–0.708) in the training and the validation cohorts, respectively. Internal and external calibration curves indicated satisfactory agreement between nomogram prediction and actual survival, and DCA indicated its clinical usefulness. Furthermore, a risk stratification system was established that was able to accurately stratify mCEC patients into three risk subgroups with significantly different prognosis.

**Conclusions:** We constructed the first nomogram and corresponding risk classification system to predict the OS of mCEC patients. These tools showed satisfactory accuracy, and clinical utility, and could aid in patient counseling and individualized clinical decision-making.

## Introduction

Worldwide, cervical cancer is the third most common malignant tumor and the fourth leading cause of cancer-related mortality in the female population (1). About 30% of cervical cancer patients present with distant metastasis at initial diagnosis, especially in developing countries (2). Despite considerable advances in the treatment, patients with distant metastasis suffer dismal prognosis, with a median survival of 8–13 months (3). Actually, metastatic cervical cancer (mCEC) is a heterogeneous disease and varies substantially in prognosis (4). The survival for mCEC is influenced by multiple factors, including age, pathological type, metastasis pattern, and treatment strategy (5, 6). Therefore, an accurate prediction of survival may benefit mCEC patients and professional doctors alike in all aspects of clinical decision-making, and render individualized treatment and surveillance possible.

Nomogram is a simple, multivariate visualization tool used in oncology to predict and quantify the survival probability of an individual patient (7). Compared to the current TNM staging system, the nomograms focuses on the individualized prognosis, and have great value in risk classification, personalized clinical management, and even clinical trial design. However, to our knowledge, almost all of the available nomograms of cervical cancer are designed for patients with localized disease, and a nomogram specifically designed for mCEC patients does not exist. Therefore, in this study we aimed to establish a prognostic nomogram and risk stratification system for mCEC patients based on the data available in the Surveillance, Epidemiology, and End Results (SEER) database, a U.S. population-based cancer database that collects the data about cancer patients from 18 registries of the U.S. and covers more than 30% of the U.S. population.

## Materials and Methods

### Patients

Information of patients initially diagnosed with mCEC between 2010 and 2014 was extracted from the SEER-18 database using SEER^*^Stat software version 8.3.5. We limited our analysis to the period of 2010–2014 as the information about site-specific metastasis is only available from 2010 and onward in the SEER database. The eligible mCEC patients included in our study were those who had only one primary malignancy; active follow-up with complete date; and complete clinicopathological information (age, race, FIGO stage, tumor grade, therapy, etc.). Since the patient information in the SEER database is de-identified and publicly available, our study was exempted from institutional review board oversight.

Variables for each patient included age, race, TNM status, pathological subtype, histology grade, T stage, N stage, distant metastatic site, treatment strategy, vital status, and survival time. In our study, TNM status was restaged according to the FIGO classification (2018 version). The distant metastasis site at diagnosis was classified as lymph node, liver, lung, bone, and brain. Surgery or radiotherapy referred to the local treatment for primary tumor. The primary endpoint of this study was overall survival (OS).

### Statistical Analyses

All eligible patients were divided randomly into the training and validation cohorts in a 7:3 split ratio. The Chi-square (χ^2^) test were used to compare the clinicopathological characteristics between the training and validation cohorts. The nomogram was developed in the training cohort as follows: First, the univariate Cox analysis was used to evaluate the ability of every variable in predicting OS. Second, variables that reached statistical significance in the univariate Cox analysis were fitted in the multivariate Cox analysis. In order to determine the independent variables that strikingly contributed to patients' prognosis, the backwards selection procedure with Akaike information criterion (AIC) score was introduced to do the variable selection. Finally, these final variables were incorporated to construct the nomogram. The nomogram adopted the 1- and 3-year OS as primary endpoints.

Validation of the nomogram was performed internally in the training cohort and externally in the validation cohort. To evaluate the predictive accuracy of the nomogram, we used the concordance index (C-index) and the receiver operating characteristic curve (ROC) analysis (8). Calibration curves (1,000 bootstrap resamples) were generated to visualize the discrimination between the predicted and actual 1- and 3-year OS. DCA, a novel algorithm, was introduced to evaluate the clinical utility of the nomogram (9).

Furthermore, a risk stratification system was developed based on the tertile of total scores in the training cohort and assigned mCEC patients into three risk subgroups, including low-, intermediate-, and high-risk groups. Kaplan-Meier analysis and log-rank test were performed to investigate the survival difference between three risk subgroups. All analyses were conducted using R software version 3.4.3. Differences were considered significant at two-sided *P*-value < 0.05.

## Results

### Patient Baseline Characteristics

Our study extracted 1,252 eligible patients diagnosed with mCEC from 2010 to 2014, including 880 patients in the training cohort and 372 patients in the validation cohort. All patients were confirmed to have distant metastasis at initial diagnosis. The median age of all patients were 56 years old with a range of 16–93 years. The majority in both cohorts were younger (≤60 years), and White. The most common pathological type was squamous cell carcinoma (65.5%). With regards to metastasis pattern, lung (31.0%) was the most frequent site, followed by bone (17.5%), liver (13.7%), and brain (2.6%). In both cohorts, most patients treated with radiotherapy or chemotherapy. Table 1 summarized the patients' baseline characteristics between the training and validation cohorts, which were comparable in our study.

**Table 1:** Characteristics of patients with metastatic cervical cancer in the training cohort and validation cohort.

**Characteristic**	**Total *N* (%)**	**Training cohort *N* (%)**	**Validation cohort *N* (%)**	***P***
	1,252 (100)	880 (70)	372 (30)	
Age (median, range)	56, 16–93	56, 19–93	55, 16–92	0.731
≤60	958 (76.5)	671 (76.2)	287 (77.2)	
>60	294 (23.5)	209 (23.8)	85 (22.8)	
Race				0.016
White	923 (73.7)	631 (71.7)	292 (78.5)	
Black	215 (17.2)	157 (17.8)	58 (15.6)	
Other	114 (9.1)	92 (10.5)	22 (5.9)	
Pathological type				0.784
Squamous cell	820 (65.5)	581 (66.0)	239 (64.2)	
Adenocarcinoma	184 (14.7)	129 (14.7)	55 (14.8)	
Other	248 (19.8)	170 (19.3)	78 (21.0)	
Histology grade				0.591
I–II	417 (33.3)	289 (32.8)	128 (34.5)	
III–IV	835 (66.7)	591 (67.2)	244 (65.6)	
T stage				0.011
T1-2	478 (38.2)	316 (35.9)	162 (43.5)	
T3-4	774 (61.8)	564 (64.1)	210 (56.5)	
N stage				0.462
N1-2	1,155 (92.3)	815 (92.6)	340 (91.4)	
N3-4	97 (7.7)	65 (7.4)	32 (8.6)	
Liver metastasis				0.603
No	1,080 (86.3)	762 (86.6)	318 (85.5)	
Yes	172 (13.7)	118 (13.4)	54 (14.5)	
Lung metastasis				0.661
No	864 (69.0)	604 (68.6)	260 (69.9)	
Yes	388 (31.0)	276 (31.4)	112 (30.1)	
Brain metastasis				0.486
No	1,219 (97.4)	855 (97.2)	364(97.8)	
Yes	33 (2.6)	25 (2.8)	8 (2.2)	
Bone metastasis				0.259
No	1,033 (82.5)	733 (83.3)	300 (80.6)	
Yes	219 (17.5)	147 (16.7)	72 (19.4)	
Metastasis numbers				0.834
0	655 (52.3)	466 (53.0)	189 (50.8)	
1	414 (33.1)	284 (32.3)	130 (34.9)	
2	153 (12.2)	109 (12.4)	44 (11.8)	
≥3	30 (2.4)	21 (2.4)	9 (2.5)	
Surgery				0.379
Not done	998 (79.7)	699 (79.4)	299 (80.4)	
Done	254 (20.3)	181 (20.6)	73 (19.6)	
Radiotherapy				
Not done	1,031 (82.3)	723 (82.2)	308 (82.8)	0.270
Done	221 (17.7)	157 (17.8)	64 (17.2)	
Chemotherapy				0.058
Not done	311 (24.8)	219 (24.9)	92 (24.7)	
Done	941 (75.2)	661(75.1)	280 (75.3)	

### Independent Prognostic Factors for Patients With mCEC

In the training cohort, the univariate Cox analysis showed that age, race, pathological type, histology grade, liver metastasis, lung metastasis, brain metastasis, bone metastasis, metastasis number, surgery, radiotherapy, and chemotherapy were identified as statistically significant prognostic factors (all *P* < 0.001) (Table 2). Among them, age (C-index = 0.593), lung metastasis (C-index = 0.594), metastasis number (C-index = 0.633), and chemotherapy (C-index = 0.641) had higher discrimination ability in predicting OS than the other factors. All these statistically significant factors were then subjected to the multivariate Cox analysis. Finally, six factors with the least value of AIC, including age, race, pathological type, histology grade, metastasis number, radiotherapy, and chemotherapy, were identified as independent prognostic factors for OS (Table 3).

**Table 2:** Univariate Cox analysis of overall survival in metastatic cervical cancer (training cohort).

**Variable**	**Reference**	**Characteristic**	**Overall survival**			
			**HR**	**95% CI of HR**	***P***	**C-index**
Age	≤60	>60	2.55	2.13–3.05	<0.001	0.593
Race	White	Black	1.410	1.147–1.733	0.001	0.529
		Others	1.196	0.909–1.574	0.201	
Pathological type	Adenocarcinoma	Squamous cell	1.496	1.128–1.985	0.005	0.539
		Other	1.097	0.861–1.397	0.454	
Histology grade	I–II	III–IV	1.260	1.056–1.504	0.010	0.538
T stage	T1-2	T3-4	0.905	0.763–1.073	0.250	0.507
N stage	N1-2	N3-4	1.041	0.768–1.412	0.796	0.500
Liver metastatic	No	Yes	2.013	1.608–2.522	<0.001	0.546
Lung metastatic	No	Yes	2.044	1.720–2.428	<0.001	0.594
Brain metastatic	No	Yes	2.579	1.644–4.046	<0.001	0.513
Bone metastatic	No	Yes	1.713	1.385–2.117	<0.001	0.542
Metastasis numbers	0	1	1.905	1.584–2.291	<0.001	0.633
	0	2	3.341	2.616–4.267	<0.001	
	0	≥3	3.221	1.910–5.431	<0.001	
Surgery	Not done	Done	0.525	0.419–0.656	<0.001	0.561
Radiotherapy	Not done	Done	0.407	0.317–0.522	<0.001	0.576
Chemotherapy	Not done	Done	0.339	0.284–0.406	<0.001	0.641

**Table 3:** Multivariate Cox analysis of overall survival in metastatic cervical cancer (training cohort).

**Variable**	**Reference**	**Characteristic**	**Overall survival**		
			**HR**	**95% CI of HR**	***P***
Age	≤60	>60	2.213	1.834–2.669	<0.001
Race	Black	White	1.490	1.117–1.988	0.006
	Black	Others	1.296	0.981–1.713	0.068
Pathological type	Adenocarcinoma	Squamous cell	1.563	1.171–2.085	0.002
	Adenocarcinoma	Other	1.263	0.984–1.622	0.066
Metastasis number	0	1	1.686	1.389–2.045	<0.001
	0	2	2.223	1.298–3.808	0.004
	0	≥3	2.686	2.087–3.456	<0.001
Radiotherapy	Not done	Done	0.603	0.467–0.779	0.007
Chemotherapy	Not done	Done	0.354	0.296–0.424	<0.001

### Nomogram Development and Validation

**Figure 1:**
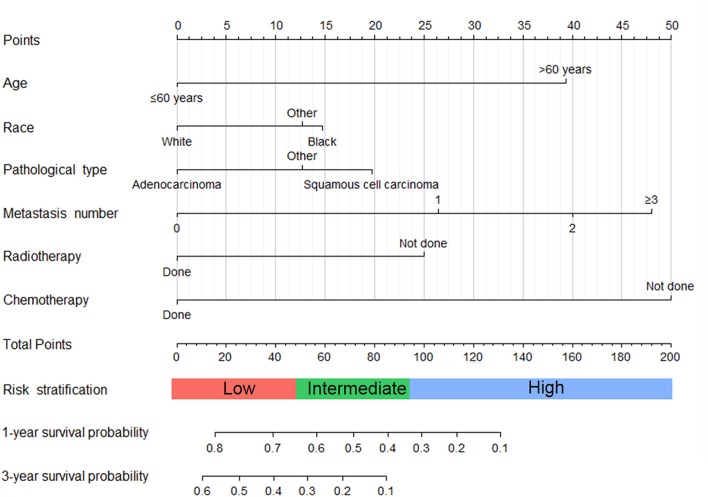
Nomogram for predicting 1- and 3-year overall survival of metastatic cervical cancer.

**Figure 2:**
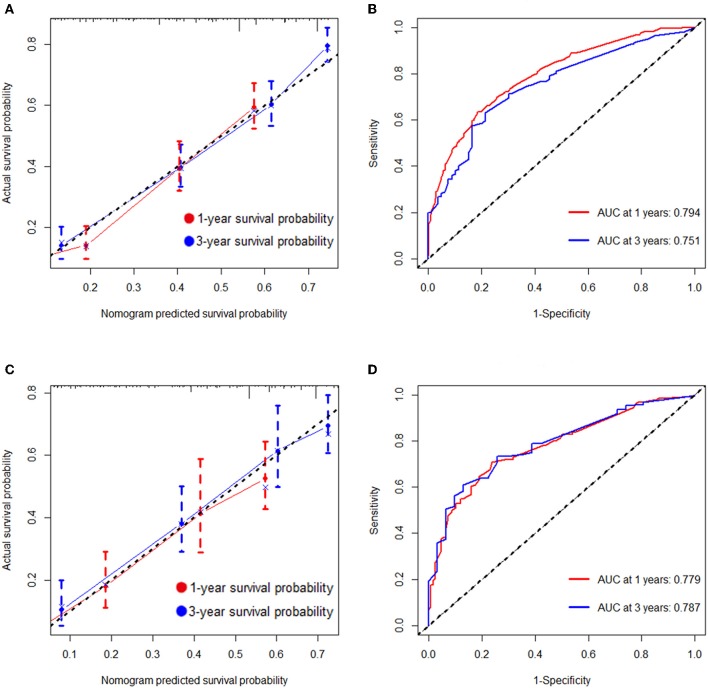
Calibration plots and ROC curves for predicting overall survival at 1- and 3- year point. **(A)** The calibration plots for predicting overall survival at 1- and 3-year point in the training cohort. **(B)** ROC curves of the nomogram for predicting overall survival at 1- and 3-year point in the training cohort. **(C)** The calibration plots for predicting overall survival at 1- and 3-year point in the validation cohort. **(D)** ROC curves of the nomogram for predicting overall survival at 1- and 3-year point in the validation cohort.

**Figure 3:**
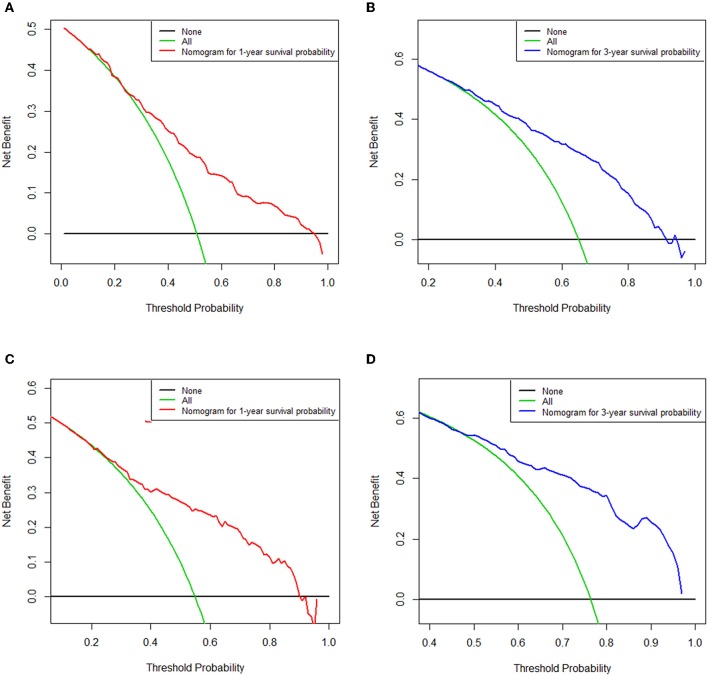
Decision curve analysis of the nomogram for predicting overall survival at 1-**(A)** and 3-year **(B)** point in the training cohort and overall survival at 1-**(C)** and 3-year **(D)** point in the validation cohort. The x-axis represents the percentage of threshold probability, whereas the y-axis represents the net benefit, calculated by adding the true positives and subtracting the false positives.

As selected by the AIC, the above-mentioned parameters were incorporated to develop the nomogram for predicting 1- and 3-year OS (Figure 1). As shown in the nomogram, chemotherapy made the largest contribution to the prognosis, followed by metastasis number and radiotherapy, interestingly. Age, pathological type showed moderate impacts on the OS, while race made the modest difference to the prognosis. The nomogram had a C-index of 0.753 (95% CI: 0.780–0.726) in the training cohort and 0.751 (95% CI: 0.794–0.708) in the validation cohort. ROC analysis showed the AUCs of this model at 1- and 3-year OS reached 0.794, 0.751 in the training cohort, and 0.779, 0.787 in the validation cohort, respectively (Figures 2B,D). The calibration curves demonstrated considerable agreement between the nomogram predicted and actual survival in both cohorts (Figures 2A,C). DCA was used to evaluate the clinical utility of the nomogram. As shown in Figure 3, the nomogram showed great positive net benefits across wide ranges of death risk in both cohorts, indicating its favorable clinical utility in predicting 1- and 3-year OS.

**Figure 4:**
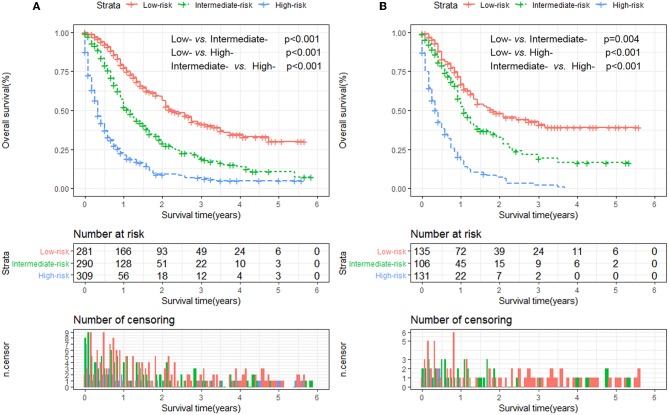
Kaplan-Meier analysis of overall survival for patients stratified by the risk stratification system in the training cohort **(A)** and validation cohort **(B)**.

Furthermore, to establish a risk stratification system based on our nomogram, we calculated the total scores for each patient in the training cohort, and then stratified the patients according to the tertile of total scores into three risk subgroups: score 0–51.0, low-risk group; score 51.1–93.0; intermediate-risk group; score 93.1–197.0, high-risk group. Each risk subgroup represented a distinct prognosis and the OS in the three subgroups was accurately separated by this system (Figures 4A,B).

## Discussion

mCEC is the end stage of cervical cancer with extremely poor survival (2). Because of its high heterogeneity, the prognosis of mCEC varies from patient to patient. Up to now, a reliable model for predicting survival in mCEC is still lacking, which leads to the difficulty in individualized clinical management and surveillance. To address this issue, we performed a real-world analysis of mCEC patients and established a prognostic nomogram and risk stratification system. The parameters in this nomogram were easily accessible from routine clinical work. Moreover, the nomogram showed excellent performance internally and externally, as indicated by C-index, calibration, ROC curves, and DCA.

The clinical value of this nomogram could be seen in the risk stratification system, which could accurately stratify mCEC patients into three risk subgroups, and might provide guidance for patient counseling and risk-adapted clinical management. For example, the National Comprehensive Cancer Network (NCCN) guidelines recommend bevacizumab in combination with systemic chemotherapy as the standard management for mCEC women (10). However, our nomogram was able to identify a high-risk subgroup who might need more intensive therapy (e.g., radiotherapy) first. For these patients, we should give them more psychological or sentimental care and encourage them to participate in clinical trials of novel drugs like immune checkpoint inhibitors. In patients with distant metastasis and squamous cell carcinoma, the 1-year OS rates were 66 and 52% in the first and second subgroups, respectively. Therefore, this new model can identify high-risk patients who had a favorable survival evaluated by the current standards. In addition, using this model, mCEC patients could be stratified in clinical trials based on the predicted prognosis, which help reduce the heterogeneity among different treatment arms (11).

In this study, six variables were identified as independent prognostic variables for OS, including age, race, pathological type, histology grade, metastasis number, radiotherapy, and chemotherapy. Among these variables, metastasis number displayed the highest discriminating power. To date, no studies have focused on the relationship between the number of metastasis sites and prognosis in mCEC patients. In our study, metastasis number was an independent prognostic factor and have a distinctly positive correlation with death risks in mCEC patients. On the other hand, the most common site of distant metastasis was lung (31.0%), followed by bone (17.5%), liver (13.7%), and brain (2.6%), which coincided with previous reports (12). Furthermore, the C-index of metastasis number was higher than that of metastasis site, indicating that the metastasis number had more powerful predictive ability. Hence, for purpose of improving survival and life quality, it is necessary to consider many aspects of prevention, early diagnosis for cervical cancer (13).

Remarkably, in our nomogram, chemotherapy had the most powerful prognostic value for OS, with the largest C-index among all factors. This result strengthened again the role of systemic chemotherapy in treating mCEC patients. To date, several clinical studies have justified multi-agent chemotherapy in patients with advanced cervical cancer, which produces a rapid response with tolerable adverse events (14, 15). Local treatment also had a role in the management of mCEC. Our nomogram indicated that radiotherapy showed significant impacts on the OS, which is consistent with previous studies (16–19).

Undeniably, our study has several limitations that should be acknowledged. The first major limitation stemmed from the lack of information about the use of targeted drugs, dosage of radiotherapy, and details of chemotherapy regimens (20). Different regimens and responses are significantly associated with survival (21, 22). In addition, lack of information about several important factors, including performance status, infection status of human papilloma virus, and comorbidities, was also one of the limitations in this nomogram. Hence, adding these predictors into future studies could refine this nomogram. Second, cancer registry data may be miscoded, which could render significant bias to our study. And we had excluded the patients who had missing data on the collected variables, leading to a selection bias (23). Third, although the nomogram and risk stratification were built using a large cohort, and validated in a split subgroup of patients, they were only developed based on the patients in the USA, which could not represent the patients in other countries. For this reason, an external validation in different countries remained necessary. Additionally, using our nomogram in the patients from randomized clinical trials would be the gold standard for validating its performance (24, 25).

In conclusion, we established the first nomogram and risk stratification system to predict the OS for patients who were initially diagnosed with mCEC. The internal and external validation suggested satisfactory performance and clinical utility of this model. However, this model should also be further evaluated in other independent population.

## Data Availability Statement

Publicly available datasets were analyzed in this study. This data can be found here: Institutional review board approval was not demanded in our study for SEER database is publicly available and we get access to it via accession number: 10165-Nov 2017.

## Author Contributions

SZ and LW contributed to the conception, design, and drafted the manuscript. SZ, XW, and ZL analyzed the data. SZ, WW, and LW contributed with a critical revision of the manuscript.

### Conflict of Interest

The authors declare that the research was conducted in the absence of any commercial or financial relationships that could be construed as a potential conflict of interest.
